# TOR Pathway-Mediated Juvenile Hormone Synthesis Regulates Nutrient-Dependent Female Reproduction in *Nilaparvata lugens* (Stål)

**DOI:** 10.3390/ijms17040438

**Published:** 2016-03-28

**Authors:** Kai Lu, Xia Chen, Wen-Ting Liu, Qiang Zhou

**Affiliations:** 1 College of Life Sciences, Fujian Agriculture and Forestry University, Fuzhou 350002, China; lukaiqw@163.com (K.L.); 18659105423@163.com (X.C.); liuwting123@163.com (W.-T.L.); 2 State Key Laboratory of Biocontrol, School of Life Sciences, Sun Yat-sen University, Guangzhou 510275, China

**Keywords:** *Nilaparvata lugens*, juvenile hormone, target of rapamycin, vitellogenesis, RNA interference

## Abstract

The “target of rapamycin” (TOR) nutritional signaling pathway and juvenile hormone (JH) regulation of vitellogenesis has been known for a long time. However, the interplay between these two pathways regulating vitellogenin (Vg) expression remains obscure. Here, we first demonstrated the key role of amino acids (AAs) in activation of Vg synthesis and egg development in *Nilaparvata lugens* using chemically defined artificial diets. AAs induced the expression of *TOR* and *S6K* (S6 kinase), whereas RNAi-mediated silencing of these two TOR pathway genes and rapamycin application strongly inhibited the AAs-induced Vg synthesis. Furthermore, knockdown of *Rheb* (Ras homologue enriched in brain), *TOR*, *S6K* and application of rapamycin resulted in a dramatic reduction in the mRNA levels of *jmt*N (juvenile hormone acid methyltransferase, JHAMT). Application of JH III on the RNAi (*Rheb* and *TOR*) and rapamycin-treated females partially rescued the *Vg* expression. Conversely, knockdown of either *jmt*N or *met* (methoprene-tolerant, JH receptor) and application of JH III had no effects on mRNA levels of *Rheb*, *TOR* and *S6K* and phosphorylation of S6K. In summary, our results demonstrate that the TOR pathway induces JH biosynthesis that in turn regulates AAs-mediated Vg synthesis in *N. lugens*.

## 1. Introduction

Nutrients are key signals regulating reproduction in insects. To be able to reproduce successfully, insects need to obtain sufficient nutrients from food resources in order to initiate and complete egg development [1,2]. However, little is known about the molecular regulatory mechanisms of the nutritional control of reproduction, especially for non-dipteran insects [3,4]. Vitellogenesis, a central process in insect egg development and maturation, involves the vitellogenin (Vg) synthesis in the fat body and its uptake by the developing oocytes via the vitellogenin receptor-mediated endocytosis [5,6,7]. In many insects, intake of proteins or amino acids (AAs) is a key trigger for the initiation of vitellogenesis. It is particularly pronounced in most anautogenous insects, in which egg development is arrested until a female takes a blood meal [8,9]. The question of how nutrients are sensed by the insects and transformed into developing eggs is very important and interesting. Both nutritional and hormonal regulation mechanisms are suggested to be involved.

The target of rapamycin (TOR), a serine/threonine kinase that is highly conserved in most eukaryotes [10,11,12], plays a pivotal role in the transduction of nutritional signals and has been proved to convey the elevated nutritional levels to stimulate Vg synthesis in the fat body and activate egg development, particularly for insects requiring a protein or amino acid feeding for the initiation of vitellogenesis [13,14]. Despite its great importance, the mechanisms of the TOR pathway in nutritional sensing are not yet fully understood in insect reproduction and most recent progress was achieved using dipteran insects, especially mosquitoes, as the research models [4]. In *Aedes aegypti*, both treatment with the TOR protein inhibitor rapamycin and RNA interference (RNAi)-mediated depletion of *TOR* gene resulted in severely decreased *Vg* gene expression in response to AAs’ stimulation in an *in vitro* fat body culture system and reduced numbers of deposited eggs *in vivo* [9]. Phosphorylation of the major downstream target of TOR, S6 protein kinase (S6K), is a critical step in the transduction of AAs’ nutritional signals to egg development in reproducing females [15,16]. As an upstream activator of TOR, the small GTPase Ras homologue enriched in brain (Rheb) is also required for transducing the AAs’ signal and activating vitellogenesis in the fat body [14]. RNAi-mediated gene depletion of either *S6K* or *Rheb* effectively disrupted *Vg* expression and blocked egg maturation after a blood meal, suggesting that the TOR pathway is indispensable for nutritionally dependent activation of Vg synthesis and oocyte maturation [14,16].

Recently, the interplay between nutritional signaling pathway and endocrine hormones involved in regulating vitellogenesis has been addressed by several studies and the regulatory mechanisms differ obviously depending on insect species with various reproductive strategies [13,17,18,19,20]. On the one hand, the TOR pathway mediates nutritional status by controlling biosynthesis and secretion of juvenile hormone (JH) and ecdysone (20E), which in turn regulate vitellogenesis. In *Blattella germanica*, the TOR pathway has been suggested to connect the nutritional signals with JH biosynthesis and as a consequence of Vg production. Transcriptional regulation of the expression of genes encoding JH biosynthetic enzymes in the corpora allata (CA) is responsible for the nutritionally regulated changes of JH levels [17]. A similar regulatory interaction between TOR nutritional signaling pathway and JH synthesis was also confirmed in the yellow fever mosquito, *A. aegypti* [13]. Conversely, JH induces Vg synthesis through regulating the expression of several genes coding for the insulin-like peptides (ILPs) in *Tribolium castaneum* [18]. Furthermore, the interplay between nutritional signaling and hormone production in regulating Vg production has been studied in a eusocial insect, *Apis mellifera* [19]. Moreover, the role of the nutritional signaling pathway in stimulation of ovarian ecdysteroidogenesis and the uptake of yolk by developing oocytes in dipterans, in which ecdysteriods are the main regulators of vitellogenesis and egg maturation, has well been established [20]. Taken together, these reports suggest that Vg expression and egg development in insects are regulated by both nutritional signaling pathway and endocrine hormones. However, the cross-talk between these two pathways that regulates vitellogenesis is complex and differs distinctly depending on insects with various reproductive strategies. More studies are urgently required in non-dipteran insects to clarify this question.

In the present study, we used the brown planthopper, *Nilaparvata lugens* (Hemiptera), a typical monophagous insect that feeds only on the rice phloem sap, as a model system because of the availability of whole genome sequence information [21,22], a large-scale expressed sequence (EST) database [23] and robust systemic RNAi sensitivity [24]. Additionally, recent studies developed a chemically defined artificial diet that could be used to investigate the role of nutrients in reproduction in *N. lugens* precisely [25,26]. Previous studies showed that JH III not only regulates Vg synthesis in the fat body, but also controls its uptake by the developing oocytes through regulating the expression of the vitellogenin receptor [27,28]. Recently, we found that AAs are indispensable for vitellogenesis and egg development in *N. lugens* [29]. In the present study, RNAi analysis, JH III topical application and chemical inhibitor experiments clearly demonstrate that the TOR nutritional signaling pathway works through JH biosynthesis to regulate Vg synthesis in response to AAs. JH, in turn, is shown to have no impact on TOR pathway in this process.

## 2. Results

### 2.1. Identification of Target of Rapamycin (TOR) Pathway Genes and Phylogenetic Analysis

The cDNA sequences of three key proteins involved in the TOR signaling pathway were identified from *N. lugens* females: Ras homolog enriched in brain (Rheb), the target of rapamycin (TOR) and S6 kinase (S6K). The *Rheb* mRNA (GenBank accession no. JX175249) from *N. lugens* encodes a protein with 182 AAs and a relative molecular mass of 20.5 kDa. *NlTOR* (GenBank accession no. JQ793898) codes for a protein comprising of 2507 amino acids (AAs) with a relative molecular mass of 283.3 kDa. It is highly conserved with 78% and 63% amino acid identity to *B. germanica* TOR and *A. aegypti* TOR, respectively. The cDNA sequence of S6K contains 1395 nucleotides, encoding 464 AAs with a highly conserved phosphorylation site “FT(p)YV” at the residue Thr-391 being analogous to threonine 389 of the human S6K.

**Figure 1:**
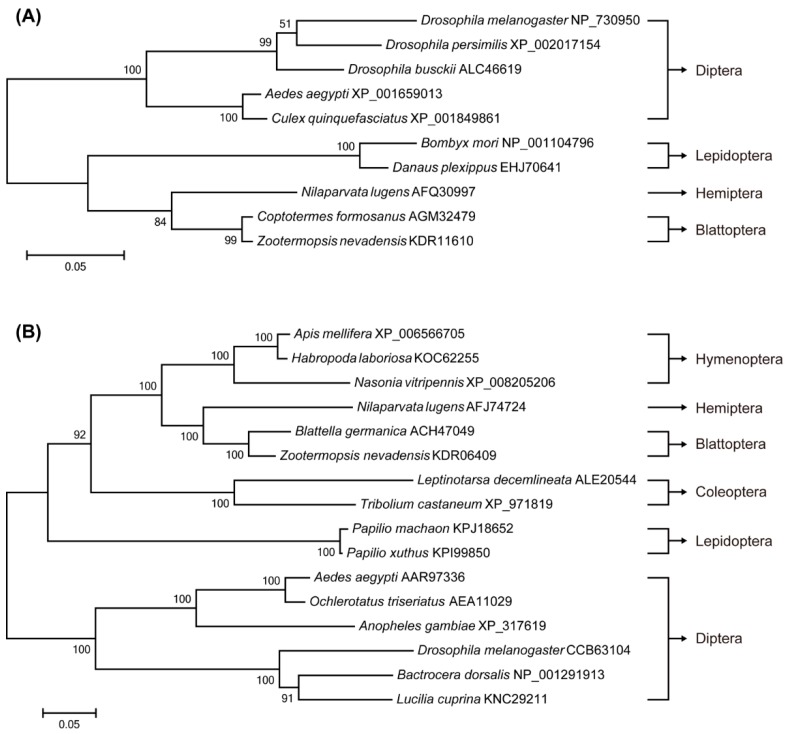

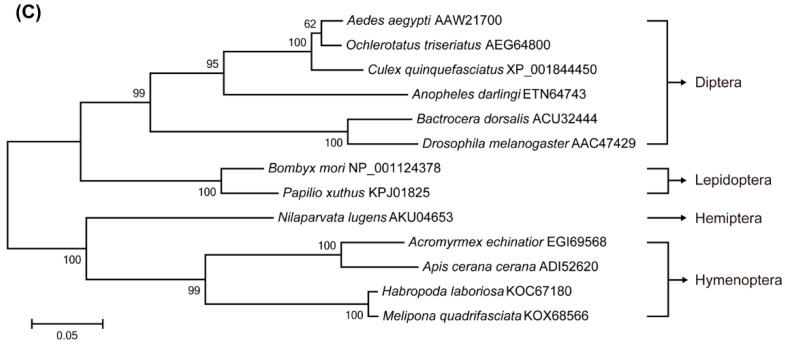
Phylogenetic analysis of Rheb (**A**), target of rapamycin (TOR) (**B**) and S6K (S6 kinase) (**C**) in insects. The tree was constructed by MEGA6 with the Maximum Likelihood method based on the amino acid sequences alignment [30]. Bootstrap test with 1000 replicates was used to analyze the evolutionary relationship and bootstrap values were shown in the cladogram. The scale bar indicates genetic distance.

To analyze the evolutionary relationship of Rheb orthologs, a Maximum-Likelihood phylogenetic tree with 1000 bootstrap replicates was constructed based on 10 Rhebs from different species (Figure 1A). Analysis of the phylogenetic relationships showed that *Nl*Rheb belongs to a single branch and the most closely related amino acid sequence to *Nl*Rheb was from a Blattoptera insect: *Zootermopsis nevadensis* (KDR11610). The phylogram based on 16 TOR protein sequences from six insect orders indicated that *Nl*TOR constitutes a monophyletic clade, and the most closely related protein sequence to *Nl*TOR was also from a Blattoptera insect: *B. germanica* (ACH47049) (Figure 1B). The evolutionary relationship of S6Ks derived from 13 species was similarly analyzed indicating that *Nl*S6K is most closely related to *Ae*S6K (*Acromyrmex echinatior*, EGI69568) (Figure 1C), which was consistent with the evolutionary relationships predicted from multiple alignments.

### 2.2. Expression of TOR Pathway Genes Is Regulated by Amino Acids (AAs) Signaling

**Figure 2:**
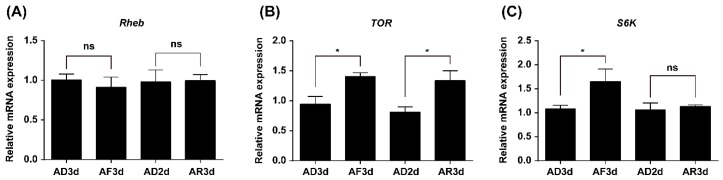
Relative mRNA levels of *Rheb* (**A**), *TOR* (**B**) and *S6K* (**C**) in the fat body of adult females upon different amino acid (AA) treatments. AD3d, newly emerged females were reared on artificial diets without AAs for three days continuously; AF3d, females were supplied with AAs for three days; AD2d, females were reared without AAs for two days; AR3d, females lacking AAs on the first and second day were supplied with AAs on the third day. Relative expression levels of *Rheb*, *TOR* and *S6K* mRNA in the fat body were detected by qRT-PCR using *β-actin* as a reference. Values were shown as mean ± SE of three independent experiments and asterisk denoted significant differences from controls (Student’s *t*-test, * denotes *p* < 0.05 and “ns” denotes *p* ≥ 0.05).

To determine whether the TOR pathway is involved in transducing AAs signals or not, qRT-PCR was used to investigate the relative expression levels of selected genes in the TOR pathway between the fat bodies of AA-deprived and -fed females. *TOR* and *S6K* expression levels were significantly decreased in the AA-deprived females (AD3d) when compared with those in AA-fed females (AF3d). Furthermore, *TOR* mRNA levels significantly increased after AA supplementation (AR3d) as compared with its expression in the AA-deprived females (AD2d). Meanwhile, *Rheb* mRNA levels showed a practically constant pattern, whether the AAs were available or not (Figure 2).

### 2.3. TOR Pathway Transduces AAs Signaling that Regulates Vitellogenin (Vg) Synthesis

**Figure 3:**
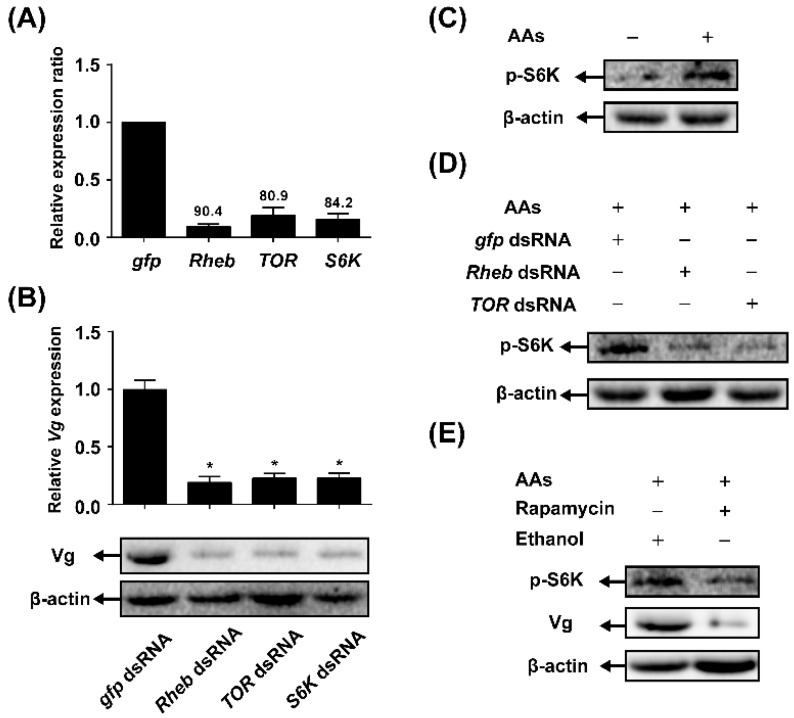
TOR pathway transduces AAs signaling that regulates vitellogenin (Vg) synthesis. (**A**) Knockdown efficiency of target genes in the fat body of the dsRNA of *gfp*, *Rheb*, *TOR* and *S6K*. The relative mRNA levels of these respective genes in control (*gfp* dsRNA) were set to 1 and the numbers above each bar indicated the percentage knockdown efficiency of corresponding gene; (**B**) Effect of the TOR pathway genes knockdown on the mRNA and protein levels of Vg (Student’s *t*-test, * denotes *p* < 0.05); (**C**) Effect of AAs on S6K phosphorylation. Western blot analysis was performed using antibody against the phospho-S6K (Thr-389); (**D**) RNAi-mediated knockdown of *Rheb* or *TOR* decreased S6K phosphorylation levels; (**E**) TOR inhibitor rapamycin effectively decreased S6K phosphorylation and Vg expression levels.

To determine whether the TOR pathway is required for AA-mediated Vg synthesis, we used a reverse genetics approach to knockdown the expression levels of TOR pathway genes *in vivo* through systemic RNAi. The mRNA levels of tested genes were determined using qRT-PCR in females injected with dsRNA of *gfp* (control) and compared with the mRNA levels of TOR pathway genes in females injected with corresponding target dsRNA. The knockdown efficiency of *Rheb*, *TOR* and *S6K* was 90.4%, 80.9% and 84.2%, respectively (Figure 3A). *Vg* mRNA levels were significantly downregulated in the RNAi treated-females. As shown in Figure 3B, the dsRNA of *Rheb*-, *TOR*- or *S6K*-injected females showed about an 80% reduction in *Vg* mRNA expression levels as compared with the control groups (dsRNA of *gfp*). Also, knockdown of the pathway genes caused a tremendous reduction in the amount of Vg protein.

Western blot analysis using anti-phosphor-S6K antibodies to measure phosphorylated S6K at position Thr 389 showed that there was a strong increase of threonine 389-phosphorylated S6K in the fat body after stimulation with AAs (Figure 3C). To determine whether TOR signaling pathway is necessary for AA-mediated phosphorylation of S6K in the *N. lugens* females or not, RNAi depletion of *Rheb* or *TOR* was applied. Phosphorylation of S6K in the fat body of adult females with *Rheb* or *TOR* depletion decreased as compared with dsRNA of *gfp* control (Figure 3D). The involvement of TOR signaling pathway was also investigated using the TOR-specific pharmacological inhibitor rapamycin. As shown in Figure 3E, the treatment of rapamycin severely suppressed S6K phosphorylation and *Vg* expression.

### 2.4. TOR Pathway Works through Juvenile Hormone (JH) Biosynthesis to Regulate Vg Synthesis

**Figure 4:**
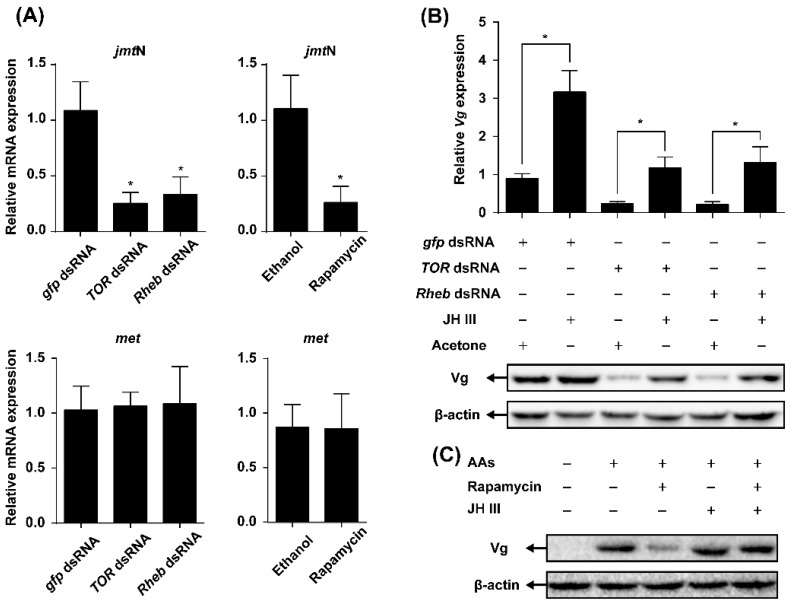
TOR pathway works through juvenile hormone (JH) biosynthesis to regulate Vg synthesis. (**A**) Relative mRNA levels of *jmt*N in the CA and *met* in the fat body of adult females after knockdown of TOR pathway genes (*TOR* or *Rheb*) and rapamycin treatment; (**B**) Relative mRNA and protein levels of Vg in the fat body of the dsRNA of *gfp*, *Rheb* and *TOR* further treated with JH III. Newly emerged females were injected with dsRNA of *gfp*, *TOR* or *Rheb* and reared for two days. After that, the insects were topically applied with JH III or acetone and provided with artificial diets for another day (Student’s *t*-test, * denotes *p* < 0.05); (**C**) Protein levels of Vg in the fat body of rapamycin-treated adult females were further topically applied with JH III.

To determine whether the influence of TOR pathway on AAs-induced Vg synthesis is through regulation of JH biosynthesis, the mRNA levels of *jmt*N in the CA and *met* in the fat body were quantified in the females injected with *TOR* or *Rheb* dsRNA or treated with rapamycin. The knockdown of *TOR* or *Rheb* by RNAi significantly decreased the mRNA expression levels of *jmt*N, but had no effect on the expression of *met* when compared with *gfp* dsRNA control. In addition, application of rapamycin caused a significant reduction in the expression levels of *jmt*N, while *met* transcript levels were not affected (Figure 4A).

To further understand the role of JH in the TOR pathway-transduced Vg synthesis, JH III was applied to the females injected with dsRNA of *TOR*, *Rheb* or *gfp*. The qRT-PCR and Western blot analyses showed that application of JH III to *TOR* or *Rheb* RNAi females partially recovered the levels of Vg mRNA and protein as compared with the acetone-treated control (Figure 4B). Similarly, Vg mRNA and protein levels in the rapamycin-treated females increased after the topical application of JH III (Figure 4C).

**Figure 5:**
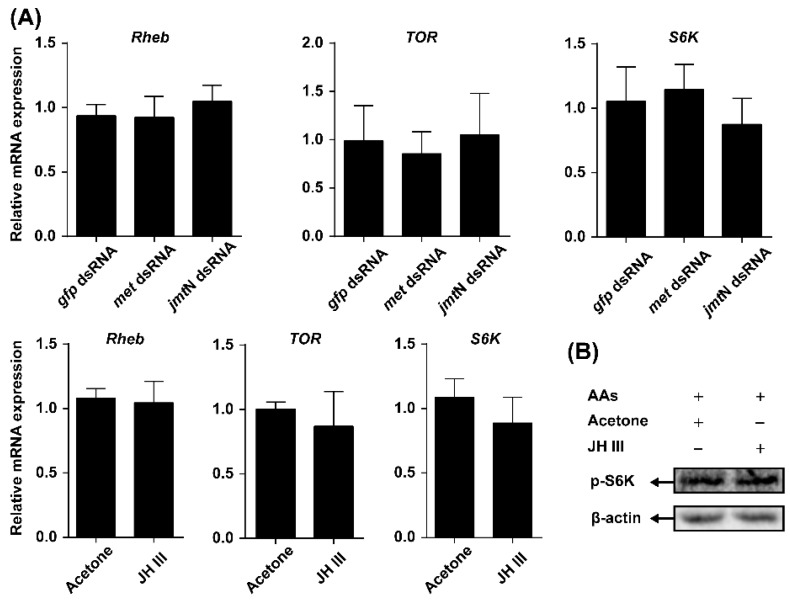
JH regulates Vg synthesis independently of TOR pathway. (**A**) RNAi-mediated knockdown of JH pathway genes (*jmt*N or *met*) and JH III application had no effect on the expression of genes related to TOR pathway (*TOR*, *Rheb* and *S6K*) in the fat body; (**B**) Total protein was extracted and phosphorylation status of S6K in the fat body was detected by Western blot.

The effects of JH on the mRNA levels of TOR pathway genes and TOR activity were also investigated. Neither *jmt*N nor *met* RNAi affected *Rheb*, *TOR* or *S6K* expression. Similarly, the mRNA levels of the TOR pathway genes were not changed after the topical application of JH III (Figure 5A). Furthermore, phosphorylation S6K levels in females treated with JH III were similar to those observed in acetone-treated control (Figure 5B).

### 2.5. TOR Pathway and JH Are Required for AA-Mediated Ovary Development and Fecundity

**Figure 6:**
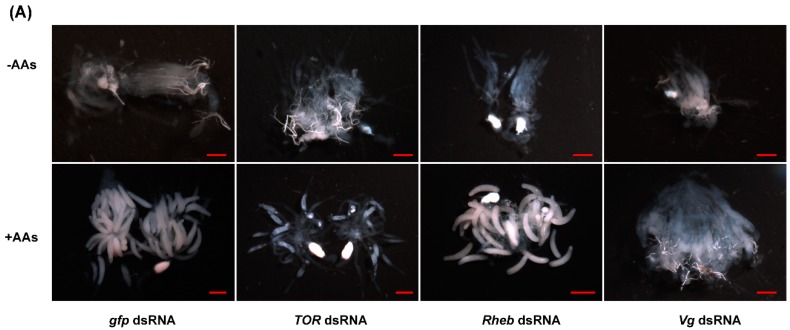

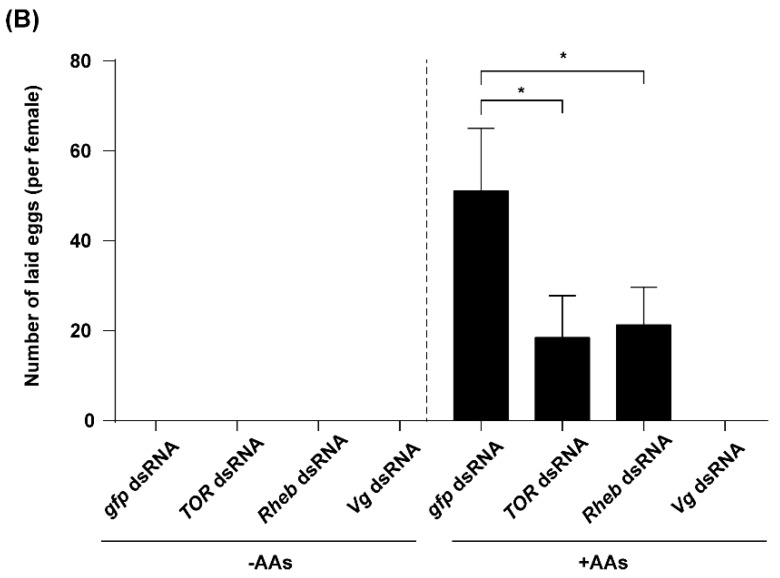
AAs, *TOR*, *Rheb* and *Vg* are required for ovary development and fecundity. (**A**) Newly emerged females were injected with dsRNA of *TOR*, *Rheb*, *Vg* or a control *gfp* and reared on artificial diets lacking AAs (−AAs) or normal diets (+AAs) for 6 days. Ovaries were dissected and photographed with a stereo microscopy SMZ18 (Nikon, Tokyo, Japan). Scale bar, 500 μm; (**B**) AAs deprivation and RNAi-mediated depletion of *TOR* or *Rheb* significantly reduced the numbers of laid eggs by females. Ten females were analyzed per group and three independent groups were evaluated. Since females lacking AAs laid no eggs, only egg numbers derived from the females feeding AAs (except for *Vg* dsRNA females) were used for statistical analysis. Student’s *t*-test was performed to determine significant differences in mean eggs numbers for knockdown females as compared with the *gfp* dsRNA-injected control group (* denotes *p* < 0.05).

Newly emerged females were injected with either dsRNA of *TOR*, *Rheb*, *Vg* or *gfp* and reared on artificial diets lacking AAs (−AAs) or normal diets (+AAs). Ovaries were dissected and photographed with a stereo microscopy. The ovarian development, in all females deprived of AAs, was completely inhibited. In the AA-fed insects, ovaries of females injected with dsRNA of *TOR*, *Rheb* or *Vg* showed a dramatic reduction in the number of mature eggs, as compared with the dsRNA *gfp*-injected females (Figure 6A). Lacking AAs rendered females sterile and no eggs were produced by the females. Besides, a significant reduction (63.8% for *TOR* and 58.32% for *Rheb*) in the number of laid eggs was observed in the dsRNA-treated females as compared with the *gfp* dsRNA control, while no eggs were found in the *Vg* dsRNA-injected females (Figure 6B).

## 3. Discussion

Our experiments clearly demonstrated the crucial role of AAs in the fecundity of *N. lugens* using a chemically defined artificial diet culture system. Previous studies proved that AAs signaling is commonly mediated by the nutrient-sensing signaling TOR pathway, and the inactivated TOR pathway is responsible for the inhibition of reproduction under limited nutrient conditions in *A. aegypti*, *B. germanica* and *T. castaneum* [3,9,17]. To investigate whether TOR pathway is involved in the AAs-mediated Vg synthesis in *N. lugens*, we first cloned the cDNAs coding for three key enzymes of the TOR pathway from this insect: *Rheb*, *TOR* and *S6K*. The transcript levels of *TOR* and *S6K* were increased significantly after AAs’ stimulation (Figure 2), whereas RNAi-mediated depletion of *Rheb*, *TOR* and *S6K* dramatically decreased *Vg* expression (Figure 3B). These results indicate that the transcription of the TOR pathway genes was significantly induced by AAs, and AAs-induced Vg synthesis was mediated by the TOR pathway. Previous reports demonstrated that AAs-dependent nutritional signaling mediates the phosphorylation of S6K, a downstream target of the TOR pathway [16]. In the present study, AAs strongly upregulated S6K phosphorylation (Figure 3C), whereas AAs supplementation showed no effects on the mRNA levels of *S6K* (Figure 2C). These results indicated that AAs were required for the activation of S6K phosphorylation: a reliable biochemical readout of TOR function [16]. To further confirm the effect of AAs on the activation of the TOR pathway, we used Western blot to analyze the phosphorylation of S6K after inhibition of TOR. RNAi-mediated gene silencing of either *Rheb* or *TOR* strongly inhibited the AAs-induced increase of S6K phosphorylation (Figure 3D). Similarly, the level of S6K phosphorylation in the fat bodies from rapamycin-treated insects was significantly decreased when compared with that of ethanol-treated controls (Figure 3E). Thus, our results suggest that the TOR pathway stimulated by AAs regulates the phosphorylation of S6K, which in turn induces *Vg* expression in *N. lugens*. It should be mentioned that RNAi of *TOR* was more effective than application of rapamycin for the inhibition of S6K phosphorylation. It was possibly due to relatively low-dose exposure to rapamycin and effective removal of *TOR* mRNA by RNAi (80.9% knockdown efficiency).

The major contribution of this study is the discovery that the TOR pathway mediates AAs-induced vitellogenesis via JH biosynthesis in a typical monophagous Hemiptera insect *in vivo*. TOR pathway or JH regulation of insect female reproduction, especially vitellogenesis, has been known for a long time, and was also confirmed by our study, but the interplay between these two pathways in regulating *Vg* expression differs depending on insects with various diet and life cycles. JH has been proved to be involved in the nutritionally dependent regulation of vitellogenesis in insect females [31,32,33,34]. The regulation of JH biosynthesis by nutrients mediated by the TOR pathway is shown in several insects [34,35]. In the mosquito *A. aegypti*, RNAi-mediated knockdown of TOR expression and application of the TOR inhibitor rapamycin severely downregulated the mRNA levels of genes coding for key enzymes involved in JH biosynthesis, with both treatments causing significant reductions in JH synthesis [13]. In the cockroach *B. germanica*, systemic depletion of *TOR* expression by RNAi also dramatically decreased the expression levels of genes coding for 3-hydroxy-3-methyl glutaryl coenzyme A (HMG-CoA) synthases and HMG-CoA reductase which was associated with the JH biosynthesis pathway in the corpora allata (CA), and also remarkably decreased mRNA levels of *Vg* in the fat body [17]. These results indicate that the TOR pathway is important to regulate JH synthesis in some kinds of insects by controlling the JH biosynthetic enzyme transcript levels. While in the red flour beetle *T. castaneum*, both nutritional signaling and JH are necessary for vitellogenesis, and JH controls the expression levels of several key genes coding for the insulin-like peptides (ILPs) involved in insulin/insulin-like growth factor signaling (IIS) pathway in this nutrient-dependent process [3,17,18,36]. In *D. melanogaster*, the IIS pathway is important to stimulate JH synthesis by regulating the expression of enzymes of the mevalonate pathway [34].

In this paper, we used a combination of *in vivo* RNAi-mediated gene depletion and pharmacology experiments to investigate the interplay between the TOR pathway and JH in regulating AAs-activated vitellogenesis in *N. lugens*. Knockdown of the TOR pathway genes (*Rheb* and *TOR*) and application of rapamycin resulted in a significant reduction in the expression levels of *jmt*N, while it had no effects on *met* (Figure 4A). These results suggest that the TOR pathway regulates JH biosynthesis rather than JH action in *N. lugens*, which is similar to those found in *B. germanica* [17]. Furthermore, application of JH III on the RNAi females (*Rheb* or *TOR*) partially recovered the *Vg* expression in response to AAs (Figure 4B). In addition, JH III alleviated the inhibitory effects of rapamycin on Vg synthesis (Figure 4C). Thus, our data confirmed previous studies in *B. germanica* [17] and *A. aegypti* [13], and demonstrated that the TOR pathway mediates AA-activated Vg synthesis through JH biosynthesis in *N. lugens*, which is different from the results observed in *T. castaneum* [18]. To further examine whether JH has regulatory effects on the TOR pathway in nutrient-mediated vitellogenesis, we used the systematic RNAi. Knockdown either *jmt*N or *met* had no effects on expression levels of the TOR pathway genes (*Rheb*, *TOR* and *S6K*). Moreover, the expression of these three genes was stable after treatment with JH III. As an activity indicator of the TOR pathway, phosphorylation of S6K was also not changed after treatment with JH III (Figure 5B). Our results revealed that the mRNA expression levels of TOR pathway genes are independent of JH. It is worth noting that post-transcriptional regulation of TOR activity by direct interaction between TOR and Rheb has been confirmed in yeast [37] and mammals [38,39]. Whether *N. lugens* TOR activity is affected by JH or not, future studies should be performed at post-transcriptional levels.

**Figure 7:**
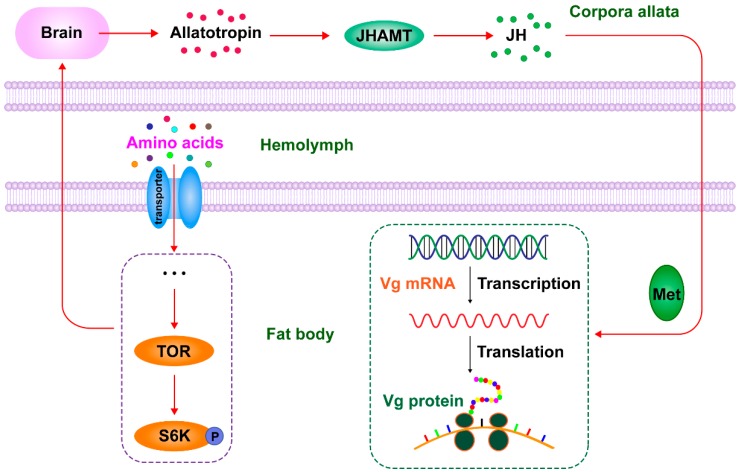
A proposed model for the TOR pathway and JH in regulating Vg synthesis responding to AAs stimulation. After feeding with normal diet, free AAs concentrations in the hemolymph are increased, the expression levels of the TOR pathway genes are elevated and S6K phosphorylation in the fat body is up-regulated. The activated TOR pathway works through some brain factors, like allatotropin, to induce JH biosynthesis in the corpora allata, and JH, in turn, activates expression of *Vg* gene in the fat body. Thus, the TOR pathway induces JH biosynthesis that, in turn, regulates AA-mediated vitellogenesis. JHAMT: juvenile hormone acid methyltransferase.

The role of brain factors in activation of JH synthesis in CA has been well established in many insects [40]. Decapitation of mosquito prevents increases of JH synthesis after a blood meal, suggesting that the brain also plays a key role in sensing the quality of nutrition and activating CA activity [41]. The factors released from the brain stimulate the CA to synthesize JH to accelerate vitellogenesis and egg maturation, and several factors are proved to be nutritionally dependent [42]. Allatotropin, a peptide hormone which is present in the brain of *A. aegypti*, shows stimulatory effects on CA activity and this in turn activates JH biosynthesis [43,44]. While, allatostain (AT) also plays a key role in transduction of the nutritional signaling that regulates JH synthesis in the CA in mosquitoes, and transcriptional regulation of the genes coding for JH biosynthetic enzymes is partially responsible for this nutritionally dependent process [40,45]. Thus, previous studies showed that the TOR signaling pathway plays an important role in sensing and transmitting nutritional signals that regulate JH synthesis in CA, and the data presented in this paper confirmed these observations and demonstrated that the TOR pathway regulates Vg synthesis by inducing expression of gene coding for JH synthetase. Therefore, we proposed a possible mechanism that the signals from the TOR pathway release AT in the brain, and then AT activates juvenile hormone acid methyltransferase (JHAMT) to methylate JH acid into JH that then binds Met and activates Vg synthesis (Figure 7).

## 4. Materials and Methods

### 4.1. Insect Rearing

The *N. lugens* strain was maintained in our laboratory as described previously [27]. Newly emerged female adults (within 12 h) were collected and reared on either normal artificial diets containing AAs (+AAs) or diets lacking AAs (−AAs, with equimolar amounts of mannitol in place of AAs) [25]. Artificial diets were held between two layers of stretched Parafilm “M” at two open ends of the glass cylinders (9.0 cm in length and 2.0 cm in diameter) and daily replenished. All artificial rearing experiments were conducted under constant conditions with 28 °C, 95% relative humidity and a light:dark (16:8 h) photoperiod in a climatic chamber.

### 4.2. Chemical Reagents and Treatments

Juvenile hormone III (Sigma-Aldrich, St. Louis, MO, USA) was dissolved in acetone. One hundred nanoliter (nL) of 1.0 ng/nL JH III was topically applied to two-day-old females and the equivalent volume of acetone (100 nL) was applied to control females. Rapamycin (Sigma-Aldrich) was dissolved in ethanol. One hundred nanoliter of 2.0 nM rapamycin was applied topically to two-day old females and the same volume of ethanol (100 nL) was topically applied to control females.

### 4.3. Sequence and Phylogenetic Analysis

The candidate cDNA sequences of *Rheb*, *TOR* and *S6K* were first identified in the genome database of *N. lugens* [22], using the homologous genes of *A. aegypti* as the templates. Full-lengths of cDNA sequences were amplified with the SMART™ RACE (rapid amplification of cDNA ends) cDNA amplification kit (BD Bioscience Clontech, Mountain View, CA, USA). All PCR products were purified using a gel extraction kit (Tiangen, Beijing, China), subcloned into pGEM-T Easy vector (Promega, Madison, WI, USA) and sequenced by Life Technologies Company (Guangzhou, China). The cDNA sequences obtained were deposited at GenBank with the corresponding accession numbers. The amino acid sequences of Rheb from 10 other species and TOR sequences from 15 insects and 12 S6K sequences were retrieved from GenBank database. Amino acid sequences were first aligned by ClustW separately, and phylogenetic trees were generated by MEGA6 using Maximum Likelihood method.

### 4.4. RNA Extraction, cDNA Synthesis and Reverse Transcriptase Quantitative Real-Time PCR (qRT-PCR)

The mRNA expression levels of the different genes in CA and fat body were analyzed by real-time PCR. Total RNA was extracted from the CA and fat body using Trizol reagent (Invitrogen, Carlsbad, CA, USA). Equal amount of total RNA treated with RNase-free DNase I was used to synthesize the first-strand cDNA using a PrimeScript RT Reagent Kit with gDNA Eraser (TaKaRa, Tokyo, Japan). Reverse transcription was carried out in 20 μL reaction mixtures containing Random 6 mers and Oligo dT primer at 37 °C for 15 min.

Quantitative real-time PCR (qPCR) was performed using the Light Cycler 480 (Roche Diagnostics, Basel, Switzerland) in 384-well plates with a SYBR Premix^Ex^ Taq Kit (TaKaRa, Tokyo, Japan). Each 10 μL qPCR reaction mixture consisted of 5 μL SYBR master mix, 0.4 μL of each primer (10 μM), 1 μL of cDNA template equivalent to 50 ng of total RNA and 3.2 μL of deionized water. The qPCR program was 95 °C for 30 s, followed by 40 cycles of 95 °C for 5 s, 60 °C for 15 s and 72 °C for 20 s. At the end of each qPCR, the dissociation curve was performed to verify the amplification specificity. To calculate the amplification efficiency of qPCR, the standard curves were generated using 10-fold serial dilutions of the cDNA pools containing high concentrations of the genes of interest. Both the qPCR efficiency (*E*) and correlation coefficient (*R*^2^) values were taken into account prior to analyzing the relative expression levels. All reactions were performed in triplicate and normalized with the reference of *N. lugens β-actin* (EU179850) for each sample. The relative mRNA expression levels (fold) were calculated according to the 2^**−**ΔΔ*C*t^ method [46]. The primers used for qRT-PCR are shown in Table S1, and primers for *Vg* (AB353856) and *β-actin* were designed as described previously [27].

### 4.5. Protein Extraction and Western Blot Analysis

Isolated fat bodies were homogenized in 0.5 mL ice-cold lysis buffer (8 M urea, 4% CHAPS, 40 mM Tris pH 8.0, 5 mM EDTA, 1 mM PMSF, 10 mM DTT) supplemented with protease inhibitor cocktail and phosphatase inhibitor mixture (Roche Diagnostics, Basel, Switzerland). After incubation for 1 h at 4 °C, lysates were cleared by centrifugation at 12,000× *g* at 4 °C for 20 min. Total protein, 30 μg, was separated by 10% SDS-PAGE and transferred to PVDF membrane (Millipore, Billerica, MA, USA). After incubation with blocking buffer (5% BSA for phosphoprotein and 5% nonfat milk for non-phosphoprotein) in Tris-buffered saline (TBS) overnight at 4 °C, the membrane was incubated with primary antibodies. The polyclonal antibodies against human S6 kinase phosphorylated on Thr 389 (p70-Thr389 S6K, Cell Signaling Technology, Danvers, MA, USA) were used to detect S6 kinase phosphoprotein. The antibodies against β-actin protein [27] was used as a loading control. After washing with TBST (Tris-buffered saline, pH 7.4, 0.5% Tween-20), the membranes were incubated with goat anti-rabbit immunoglobulin G horseradish peroxidase-linked secondary antibodies (Sigma-Aldrich, St. Louis, MO, USA) in the same blocking buffer for 1 h at room temperature. The bands were visualized with chemiluminescent substrate ECL (Pierce, Rockfod, IL, USA) and photographed with the GBOX-Chemi XT4 (Syngene, Cambridge, UK).

### 4.6. RNA Interference

For synthesis of double-stranded RNA (dsRNA), PCR primers were designed against specific regions of *N. lugens met* (KJ690934, 499 bp), *jmtN* (KP769805, 548 bp), *TOR* (JQ793898, 563 bp), *S6K* (KP769804, 503 bp), *Rheb* (JX175249, 349 bp) and *Vg* (AB353856, 538 bp) with T7 RNA polymerase promoter linked to both 5′ and 3′ ends. The PCR products were purified using a gel extraction kit (Tiangen, Beijing, China), and dsRNAs were produced by means of *in vitro* transcription with T7 polymerase using a T7 RiboMAX™ Express RNAi System (Promega, Madison, WI, USA). By using the same technique, dsRNA against *gfp* (green fluorescent protein) gene (ACY56286, 542 bp) was generated as a negative control [47]. All dsRNAs were precipitated with ammonium acetate/ethanol, and dissolved in diethyl pyrocarbonate (DEPC)-treated water to a final concentration of 4.5 μg/μL and stored at −80 °C. In each knockdown experiment, newly emerged females were carbon dioxide anaesthetized and injected with 23 nL dsRNA (about 100 ng) using a Nanoject II microinjection device (Drummond Scientific, Broomall, PA, USA) [48,49]. Knockdown efficiency of gene expression in the RNAi females was calculated as the ratio of mRNA expression levels between target dsRNA-injected and control *gfp* dsRNA-injected females. The primers used for dsRNA synthesis were shown in Table S1, and primers for *Vg* and *gfp* dsRNAs were designed as described previously [27].

### 4.7. Ovary Dissection and Fecundity Analysis

Newly emerged females were injected with either dsRNA of *TOR*, *Vg* or a control *gfp* and reared on artificial diets lacking AAs (−AAs) or normal diets (+AAs) for 6 days. Ovaries were dissected in cold phosphate buffered saline (PBS) and photographed with a stereo microscopy SMZ18 connected to a DS-Fi2 digital camera (Nikon, Tokyo, Japan). Ovaries from at least 15 females for each treatment were analyzed.

Each dsRNA-injected female was matched with two males and put into an oviposition apparatus (glass cylinder with 4.0 cm in length and 2.0 cm in diameter) for fecundity analysis. Artificial diets were held between two layers of stretched Parafilm “M” at one open end of the cylinder and oviposition mediums (5% sucrose with 4 μM salicylic acid) were put on another end of the cylinder [25]. Artificial diets and oviposition mediums were daily replenished, meanwhile, the laid eggs were counted under a stereo microscopy SMZ18 (Nikon, Tokyo, Japan) for fifteen days. Ten females were analyzed per group and three independent groups were evaluated.

### 4.8. Statistical Analysis

SPSS 18.0 software (SPSS Inc., Chicago, IL, USA) was used for statistical analysis. Normality of data variances was examined by the *Shapiro–Wilk* test. For data which showed normal and homogenous distribution, *t*-test was performed for comparison of means between two variables. Significant differences were considered at *p* < 0.05. All values are expressed as mean ± SE.

## 5. Conclusions

In summary, our results suggest that AAs are a key nutritional signal in Vg synthesis in *N. lugens*. The TOR pathway induces JH biosynthesis that in turn regulates vitellogenin synthesis in response to AAs (Figure 7), thus providing a partial answer to the question on the cross-talk between nutritional signals and hormones that regulate vitellogenesis in non-dipteran insects. In addition, it is worth noting that 20E and IIS signaling pathways regulate Vg expression in the fat body both directly and indirectly [50,51]. To date, at least four different pathways have been found to be involved in regulating nutritionally dependent vitellogenesis: JH, 20E, IIS and TOR nutritional signaling pathways [52]. The challenge for future study is to understand the cross-talk between these pathways.

## Supplementary Materials

Supplementary materials can be found at http://www.mdpi.com/1422-0067/17/4/438/s1.
