# Supplementary material for: TOR Pathway-Mediated Juvenile Hormone Synthesis Regulates Nutrient-Dependent Female Reproduction in Nilaparvata lugens (Stål)
Source: Int J Mol Sci. 2016 Mar 28;17(4):438. doi: 10.3390/ijms17040438 (PMC4848894; doi:10.3390/ijms17040438)
Supplement: Supplementary file 1 [file ijms-17-00438-s001.pdf]

Supplementary Materials: TOR Pathway-Mediated Juvenile Hormone Synthesis Regulates Nutrient-Dependent Female Reproduction in *Nilaparvata lugens* (Stål)

Kai Lu, Xia Chen, Wen-Ting Liu and Qiang Zhou

Table S1. Primers used in this study. TOR: target of rapamycin; S6K: S6 kinase.

Purpose	Primer Name	Primer Sequence (5′-3′)
qPCR analysis	Q-*TOR*-F	AACGCCATGGAGGTGACAGG
	Q-*TOR*-R	ATGAGGCGCCAGTTGAGCAG
	Q-*S6K*-F	AGGACACTGCGCACACCAAG
	Q-*S6K*-R	CAATTCTCCGCCGCTCAGAT
	Q-*Rheb*-F	ACGGCGGGTCAGGATGAGTA
	Q-*Rheb*-R	TCGAAGGACTTGGATGAGGTGA
	Q-*jmt*N-F	GAACCTGCAGGCCAAACACA
	Q-*jmt*N-R	ACCACTCGGTTGGGCTGAAT
	Q-*met*-F	AGTGGCAGCGAGCGATGATT
	Q-*met*-R	TGAGGCGCAGCAAAAAGGAG
dsRNA synthesis	*TOR*-Fi	CAGTGGCCCGAGGTCTACGA
	TOR-Ri	TCCCACGCCTGGTTCAAGTC
	TOR-T7Fi	TAATACGACTCACTATAGGGCAGTGGCCCGAGGTCTACGA
	TOR-T7Ri	TAATACGACTCACTATAGGGTCCCACGCCTGGTTCAAGTC
	S6K-Fi	CATTGTGCGCAACCAGAAGG
	S6K-Ri	TTTCGGCAGTGAAGGGAGGA
	S6K-T7Fi	TAATACGACTCACTATAGGGCATTGTGCGCAACCAGAAGG
	S6K-T7Ri	TAATACGACTCACTATAGGGTTTCGGCAGTGAAGGGAGGA
	Rheb-Fi	GTTGATACGGCGGGTCAGGA
	Rheb-Ri	GCATGTTTCCGTTGGCCTTT
	Rheb-T7Fi	TAATACGACTCACTATAGGGGTTGATACGGCGGGTCAGGA
	Rheb-T7Ri	TAATACGACTCACTATAGGGGCATGTTTCCGTTGGCCTTT
	*jmt*N-Fi	CTCCAGGCCATTGTCCCTCA
	*jmt*N-Ri	TTGGCCTGCAGGTTCTTTGG
	*jmt*N-T7Fi	TAATACGACTCACTATAGGGCTCCAGGCCATTGTCCCTCA
	*jmt*N-T7Ri	TAATACGACTCACTATAGGGTTGGCCTGCAGGTTCTTTGG
	met-Fi	GACCCAAGCCACCCTCCAAG
	met-Ri	TCCCCATCGTCAGCCAACTC
	met-T7Fi	TAATACGACTCACTATAGGGGACCCAAGCCACCCTCCAAG
	met-T7Ri	TAATACGACTCACTATAGGGTCCCCATCGTCAGCCAACTC
